# *Genlisea hawkingii* (Lentibulariaceae), a new species from Serra da Canastra, Minas Gerais, Brazil

**DOI:** 10.1371/journal.pone.0226337

**Published:** 2020-01-15

**Authors:** Saura Rodrigues Silva, Bartosz Jan Płachno, Samanta Gabriela Medeiros Carvalho, Vitor Fernandes Oliveira Miranda

**Affiliations:** 1 São Paulo State University (Unesp), School of Agricultural and Veterinarian Sciences, Laboratory of Plant Systematics, Jaboticabal, SP, Brazil; 2 Department of Plant Cytology and Embryology, Institute of Botany, Faculty of Biology, Jagiellonian University in Kraków, Kraków, Poland; Università di Pisa, ITALY

## Abstract

*Genlisea hawkingii*, which is a new species of *Genlisea* subgen. *Tayloria* (Lentibulariaceae) from *cerrado* in southwest Brazil, is described and illustrated. This species has been found in only one locality thus far, in the Serra da Canastra, which is located in the Delfinópolis municipality in Minas Gerais, Brazil. The new species is morphologically similar to *Genlisea violacea* and *G*. *flexuosa*, but differs from them in having a corolla with a conical and curved spur along with sepals with an acute apex and reproductive organs that only have glandular hairs. Moreover, it is similar to *G*. *uncinata*’s curved spur. *G*. *hawkingii* is nested within the subgen. *Tayloria* clade as a sister group to all the other species of this subgenus. Therefore, both morphological and phylogenetic results strongly support *G*. *hawkingii* as a new species in the subgen. *Tayloria*.

## Introduction

*Genlisea* A.St.-Hil. is a mainly Neotropical genus that includes ca. 30 species of carnivorous plants [1,2]. The genus belongs to the family Lentibulariaceae [3] and has recently aroused special interest for its tiny genomes, such as that of *Genlisea tuberosa*, which is the smallest genome that has ever been found in angiosperms [4]. *Genlisea* was first split into two sections and separated according to fruit dehiscence: *Genlisea* sect. *Genlisea*, which has a circumscissile dehiscence, and *G*. sect. *Tayloria*, whose fruits open through longitudinal slits [5]. In more recent times, Fromm-Trinta’s *Tayloria* [5] was raised to the subgeneric rank [6]. Both subgenera are considered to be monophyletic groups according to molecular phylogenetic data [1,7].

The *Genlisea* subgen. *Tayloria* includes eight species all endemic to Brazil [1,8,9], and chiefly colonizing the highlands in Minas Gerais. Particularly, most of the species are from Serra do Espinhaço in Minas Gerais [10]. They are mainly annual species, though some are known to be perennials [1].

During field trips, we discovered *Genlisea* plants that did not seem to belong to any species that had been previously described. Although detailed comparisons revealed similarities between *G*. *violacea* and *G*. *flexuosa*, significant differences were also observed.

Here, we describe a new species of *Genlisea*, endemic to the Serra da Canastra region of the Delfinópolis municipality in Minas Gerais. We also provide a full taxonomic description, comments, photographs and illustrations for the new species as well as a phylogenetic analysis based on the chloroplast DNA regions of *Genlisea* subgen. *Tayloria*, in order to assess the phylogenetic position of the new taxon.

## Material and methods

### Plant material

We collected the specimens in Serra da Canastra in the Delfinópolis municipality, Minas Gerais, Brazil. The individuals were pressed for dry vouchers and then fixed in a FAA70 (Formaldehyde-Glacial Acetic Acid-Ethyl Alcohol 70%) solution for the morphological analyses, which were performed using a stereomicroscope and light microscope. In addition, some specimens were fixed in a mixture of 5% glutaraldehyde with 2.5% formaldehyde in a 0.006-M cacodylate buffer (pH 7.2) and the morphological analyses were performed using a scanning electron microscope (SEM). For the SEM, the fixed material was dehydrated, subjected to critical drying point using liquid CO_2_, and sputter-coated with gold and digital photos of the analyzed material were taken with a Hitachi S-4700 scanning electron microscope (Hitachi, Tokyo, Japan). The morphological traits were measured for all of the collected specimens using a digital caliper and a Leica® stereomicroscope using the Leica® IM50 program, calibrated for the magnification used. The comparison between similar species was based on field observations, herbarium specimens, and information that has been gathered from species’ protologues and monograph on *Genlisea* [1]. The morphological terminology and structure of the description follow [1]. The herbarium abbreviations that are cited in the text follow [11].

### Molecular analyses

For the phylogenetic analyses, DNA was extracted from floral axes and flowers of the new *Genlisea* species using a Qiagen® DNeasy Plant Mini Kit, thus avoiding any contamination from other organisms, such as prey, from the photosynthetic leaves and carnivorous traps. The chloroplast DNA regions were amplified and sequenced from the *rps*16 region using the primers RPSF 5’-GTGGTAGAAAGCAACGTGCGACTT-3’ and RPSR2 5’-TCGGGATCGAACATCAATTGCAAC-3’ [12], which were used in previous *Genlisea* studies [7] and *mat*K using the primers 3F-KIM 5’-CGTACAGTACTTTTGTGTTTACGAG-3’ and 1R-KIM 5’-ACCCAGTCCATCTGGAAATCTTGGTTC-3’, which were designed for plant DNA barcoding studies by Kim [13]. Reaction conditions for the *rps*16 region included denaturation at 95ºC for 5 min followed by 29 cycles of 30 sec at 95ºC, 30 sec at 52ºC and 2 min at 72ºC, followed by a final extension at 72ºC for 5 min. For the *mat*K region, the reaction was performed with denaturation at 94ºC for 1 min followed by 35 cycles of 40 sec at 94ºC, 20 sec at 52ºC and 50 sec at 72ºC, followed by a final extension at 72ºC for 5 min. All amplifications were performed in a PTC-100 (MJ Research) thermal cycler. The sequences were amplified for both strands (forward and reverse) and the consensus sequences were assembled using BioEdit v. 7.0.5 [14].

### Phylogenetic analyses

Using the sequences of other *Genlisea* species, *Utricularia gibba* and *Pinguicula alpina* (Table 1), which are available in GenBank (NCBI), the sequences were aligned using the online version of MAFFT v. 7 [15]. The *Pinguicula* and *Utricularia* sequences were used as the outgroup. The matrices were trimmed according to the *Genlisea* amplified sequences and the genes were concatenated into a single matrix. All gaps were treated as missing. Two methods were used for the phylogenetic reconstruction: maximum likelihood (ML) using RAxML v. 8 [16] software and Bayesian inference (BI) using Mr. Bayes v. 3.2.2 [17]. For the BI, 5×10^7^ generations were calculated until the standard deviation reached a value below 0.01 using two runs with four chains. In each run, the trees were sampled every 1,000 generations at a sample frequency of 100. The first 25% of the trees that were initially produced were discarded as burn-in. The BI analyses was conducted using the TVM+G model and was calculated using jModeltest v. 2 software [18] following the Akaike information criterion [19]. The ML was run using the GTRGAMMA model and the bootstrap support values were generated with 1,000 pseudo-replicates using a rapid bootstrap algorithm [20] implemented in RAxML. All of the phylogenetic and model test analyses were conducted using the CIPRES Science Gateway online platform [21]. The trees were edited using TreeGraph v. 2 [22].

**Table 1:** Genbank accession numbers of the taxa that were used in this study. The sequences indicated by an * were newly generated. The species names of *G*. *flexuosa* and *G*. *metallica* were changed from the Genbank reference according to [1].

Species	*Genlisea* subgenus	*mat*K	*rps*16 region
*Genlisea africana* Oliv.	*Genlisea*	FN641702	FN641735
*Genlisea aurea*^1^ A.St.-Hil.	*Genlisea*	FN641695	FN641745
*Genlisea aurea*^2^ A.St.-Hil.	*Genlisea*	FN641714	FN641743
*Genlisea aurea*^3^ A.St.-Hil.	*Genlisea*	FN641693	FN641746
*Genlisea aurea*^4^ A.St.-Hil.	*Genlisea*	FN641694	FN641744
*Genlisea barthlottii* S.Porembski, Eb.Fisch. & B.Gemmel	*Genlisea*	FN641704	FN641732
*Genlisea filiformis*^1^ A.St.-Hil.	*Genlisea*	FN641691	FN641748
*Genlisea filiformis*^2^ A.St.-Hil.	*Genlisea*	FN641690	FN641749
*Genlisea flexuosa*^1^ Rivadavia, A.Fleischm. & Gonella	*Tayloria*	FN641717	FN641720
*Genlisea flexuosa*^2^ Rivadavia, A.Fleischm. & Gonella	*Tayloria*	FN641713	FN641719
*Genlisea glabra* P.Taylor	*Genlisea*	FN641692	FN641747
*Genlisea glandulosissima*^1^ R.E.Fr.	*Genlisea*	FN641699	FN641739
*Genlisea glandulosissima*^2^ R.E.Fr.	*Genlisea*	FN641700	FN641738
*Genlisea guianensis*^1^ N.E.Br.	*Genlisea*	FN641697	FN641739
*Genlisea guianensis*^2^ N.E.Br.	*Genlisea*	FN641696	FN641775
*Genlisea hawkingii* S.R.Silva, B.Płachno & V.Miranda	*Tayloria*	MN453285*	MN453284*
*Genlisea hispidula*^1^ Stapf	*Genlisea*	FN641705	FN641731
*Genlisea hispidula*^2^ Stapf	*Genlisea*	AF531815	FN641730
*Genlisea lobata* E.Fromm-Trinta	*Tayloria*	FN641711	FN641723
*Genlisea margaretae*^1^ Hutch.	*Genlisea*	FN641701	FN641736
*Genlisea margaretae*^2^ Hutch.	*Genlisea*	AF531816	FN641737
*Genlisea metallica* Rivadavia & A.Fleischm.	*Tayloria*	FN641712	FN641721
*Genlisea pygmaea* A.St.-Hil.	*Genlisea*	FN641686	FN641754
*Genlisea repens* Benj.	*Genlisea*	FN641689	FN641751
*Genlisea roraimensis* N.E.Br.	*Genlisea*	AF531817	FN641750
*Genlisea sanariapoana* Steyerm.	*Genlisea*	FN641698	FN641740
*Genlisea stapfii* A.Chev.	*Genlisea*	AF531818	FN641733
*Genlisea subglabra* Stapf	*Genlisea*	FN641706	FN641729
*Genlisea subviridis* Hutch.	*Genlisea*	FN641703	FN641734
*Genlisea uncinata* P.Taylor	*Tayloria*	AF531819	FN641718
*Genlisea violacea*^1^ A.St.-Hil.	*Tayloria*	FN641716	FN641728
*Genlisea violacea*^2^ A.St.-Hil.	*Tayloria*	FN641707	FN641726
*Genlisea violacea*^3^ A.St.-Hil.	*Tayloria*	FN641715	FN641724
*Genlisea violacea*^4^ A.St.-Hil.	*Tayloria*	FN641708	FN641727
*Genlisea violacea*^5^ A.St.-Hil.	*Tayloria*	FN641709	FN641725
*Genlisea violacea*^6^ A.St.-Hil.	*Tayloria*	FN641710	FN641722
*Pinguicula alpina* L.	-	AF531783	AF482544
*Utricularia gibba* L.	*-*	MH552396	AF482572

### Nomenclature

The electronic version of this article in Portable Document Format (PDF) in a work with an ISSN or ISBN will represent a published work according to the International Code of Nomenclature for algae, fungi, and plants, and hence the new names contained in the electronic publication of a PLOS article are effectively published under that Code from the electronic edition alone, so there is no longer any need to provide printed copies.

In addition, new names contained in this work have been submitted to IPNI, from where they will be made available to the Global Names Index. The IPNI LSIDs can be resolved and the associated information viewed through any standard web browser by appending the LSID contained in this publication to the prefix http://ipni.org/. The online version of this work is archived and available from the following digital repositories: PubMed Central and LOCKSS.

### Ethics statement

Because the samples were not collected from a conservation unit, no collection permits were required. Thus, the field studies did not involve endangered or protected species.

## Results

### Taxonomic treatment

**Fig 1:**
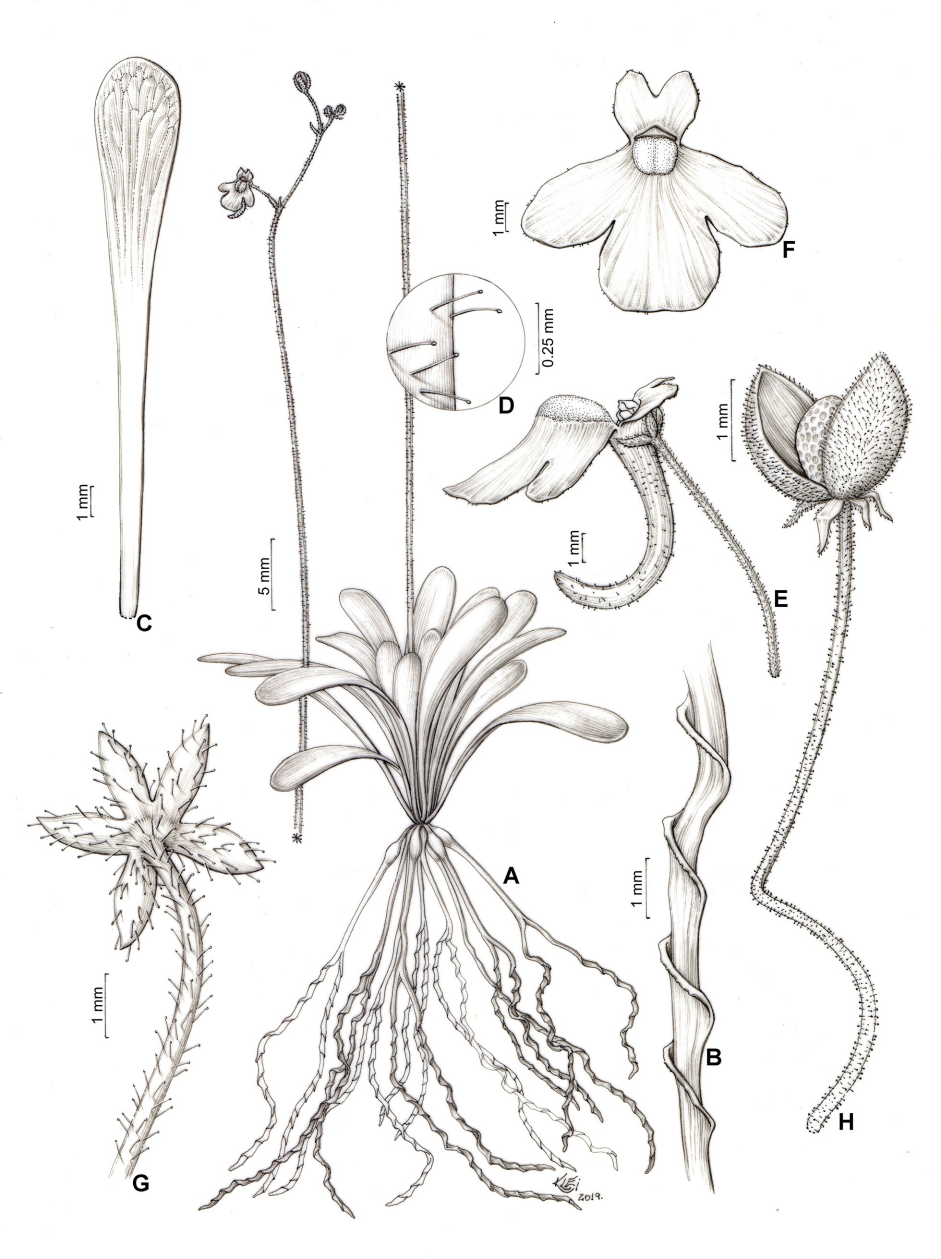
Genlisea hawkingii. A. Habit. B. Rhizophyll. C. Glabrous leaf. D. Glandular capitate hairs. E. Open corolla, lateral view. F. Corolla, front view. G. Calyx. H. Capsule.

**Fig 2:**
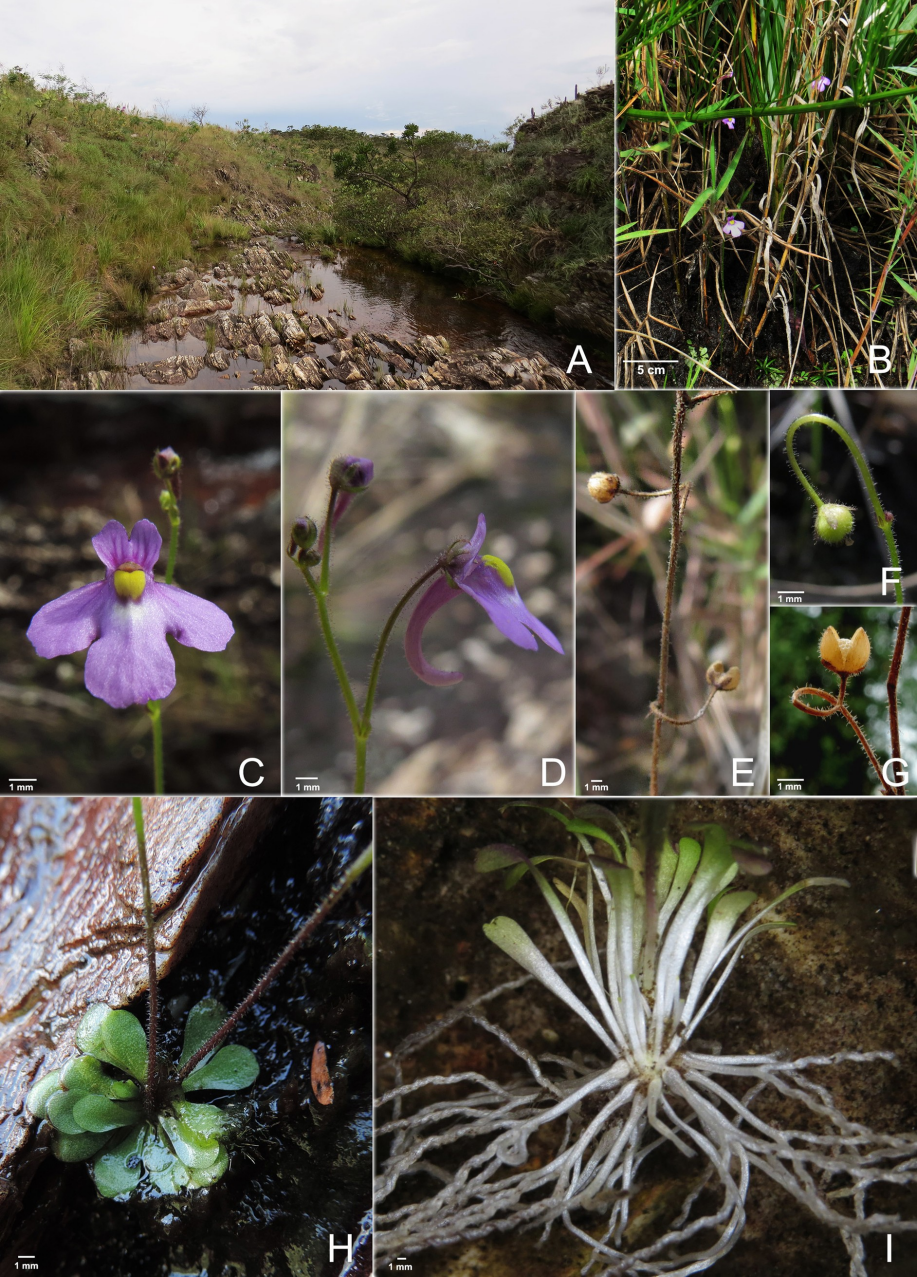
Genlisea hawkingii. A. Habitat. B. Habit of the plant in Delfinópolis, Minas Gerais (Brazil). C. Corolla, front view. D. Corolla, lateral view. E. Dry infructescence. The pedicel twist upward. F. Immature fruit. G. Mature fruit. Pedicels bent upward. H. A rosette with two scapes. I. A rosette with photosynthetic leaves (above) and rhizophylls (below).

**Fig 3:**
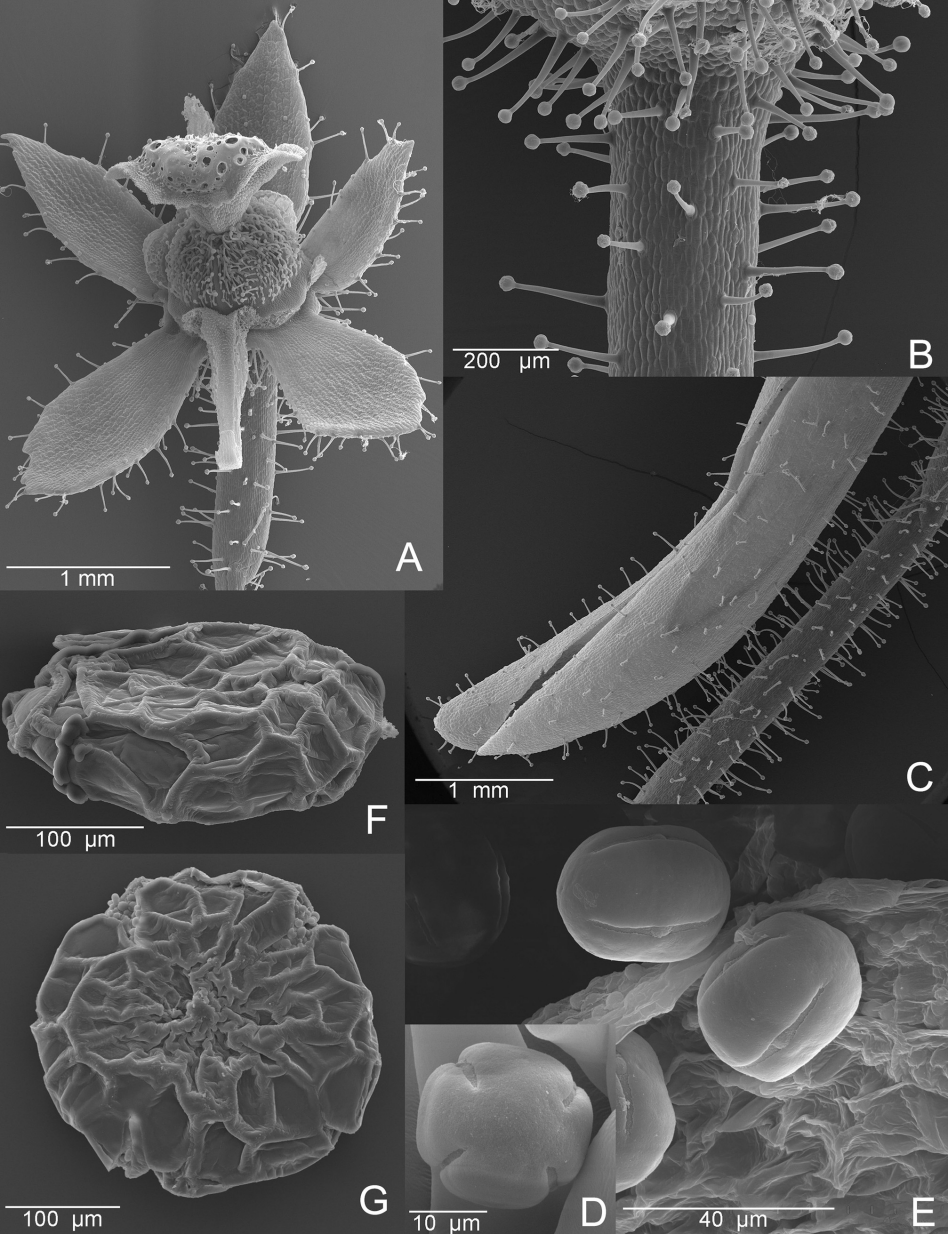
SEM images of *Genlisea hawkingii*. A. Flower, corolla and stamens removed, showing the gynoecium and sepals. B. Distal part of a pedicel. C. Cut spur. Note images A-C showing only glandular capitate hairs. D-E. Pollen grains (D–polar view, E–equatorial view). F-G. Seeds (F–Lateral view, G–Front view).

*Genlisea hawkingii* S.R.Silva, B.J.Płachno & V.Miranda, *sp*. *nov*. [urn:lsid:ipni.org:names: 77203166–1] (Figs 1–3).

### Diagnosis

Similar to *Genlisea violacea* A.St.-Hil. and *G*. *flexuosa* Rivadavia, A.Fleischm. & Gonella, but it is distinct for the dark green leaves having a glabrous lamina and the flower that has a long conical spur with a curved apex, acute sepals apex and reproductive organs that are exclusively covered with glandular hairs.

### Type

BRAZIL. Minas Gerais: Delfinópolis, Serra da Canastra, near “Casinha Branca”, *cerrado*, sandy soil, rare, 02 March 2019, *V*.*F*.*O*. *Miranda et al*. 2359 (HOLOTYPE: JABU!; ISOTYPES: INPA!, RB!).

### Description

*Habit* small terrestrial annual herb, up to 30 cm tall; lax rosette of ca. 20 leaves. *Leaves* numerous, spatulate, lamina obovate, green or dark green, with apex rounded, up to 12 mm long and 1.5–3.0 mm wide, glabrous, *petiole* 4-5(8) mm long, flattened, widened into the lamina. *Rhizophylls* many, uniform, up to 53 mm long, the largest part consisting of the trap arms; stalk short, up to 2.8 mm long and 0.5 mm wide; vesicle narrowly cylindrical, up to 1.3–3.6 mm long up to 1.3 mm wide; neck 3.5–6.8 mm long and up to 0.7 mm wide; trap arm up to 30 mm in length and width with ca. 24 twists. *Inflorescence* lax raceme, many-flowered, unbranched, up to 27 cm long and 0.4 mm thick diameter (0.6 mm near the base); scape, bracts, bracteoles and calyx densely covered by stalked glandular capitate hairs 0.15–0.2 mm long. *Scapes* up to 3, *bracts* narrowly obovate to linear-triangular, 1.4 mm long and 0.5 mm wide; *bracteoles* subulate, up to 1.1 mm long and 0.3 mm wide. *Flowers* 1–7 per inflorescence distally inserted on the branched axis; *Pedicels* slightly curved at anthesis, 7–14 mm long, 0.4 mm diameter, during fructification pedicels are elongated, up to 18 mm long and curved downwards, and densely covered with glandular capitate hairs. *Sepals* subequal, lanceolate with acute apex, about 0.7 mm long and 0.6 mm wide, densely covered with glandular capitate hairs. *Corolla* 7–10 mm long (excluding the spur), pale lavender to lilac, with two yellow ridges forming a round marking at the base of the lower lip, a few individuals have a white blotch in the lower lip, the upper lip often has darker purple streaks along the nerves, margins with glandular capitate hairs; *upper lip* ovate has one third divided in two lobes, each lobe ca. 1.5 mm wide, with apex cleft; *lower lip* up to 7 mm long and 10 mm wide, trilobate, lobes subequal, median lobe 3 mm wide, lateral lobes 2.5 mm wide, short with apex obtuse to slightly retuse; s*pur* conical, apex curved, straightening towards the apex, longer than the lower lip, 6 mm long and 1.3 mm in diameter at the base and 0.3 m in diameter at the apex, covered with glandular capitate hairs. *Capsule* globose, (1.7)2-3 mm, densely covered with only glandular hairs, opening longitudinally bivalvate. *Seeds* prismatic, 0.20–0.26 mm long and 0.24–0.31 mm wide, 0.14 mm high, testa reticulate with cells polygonal, isodiametric, anticlinal boundaries straight and raised periclinal walls tabular.

### Distribution

**Fig 4:**
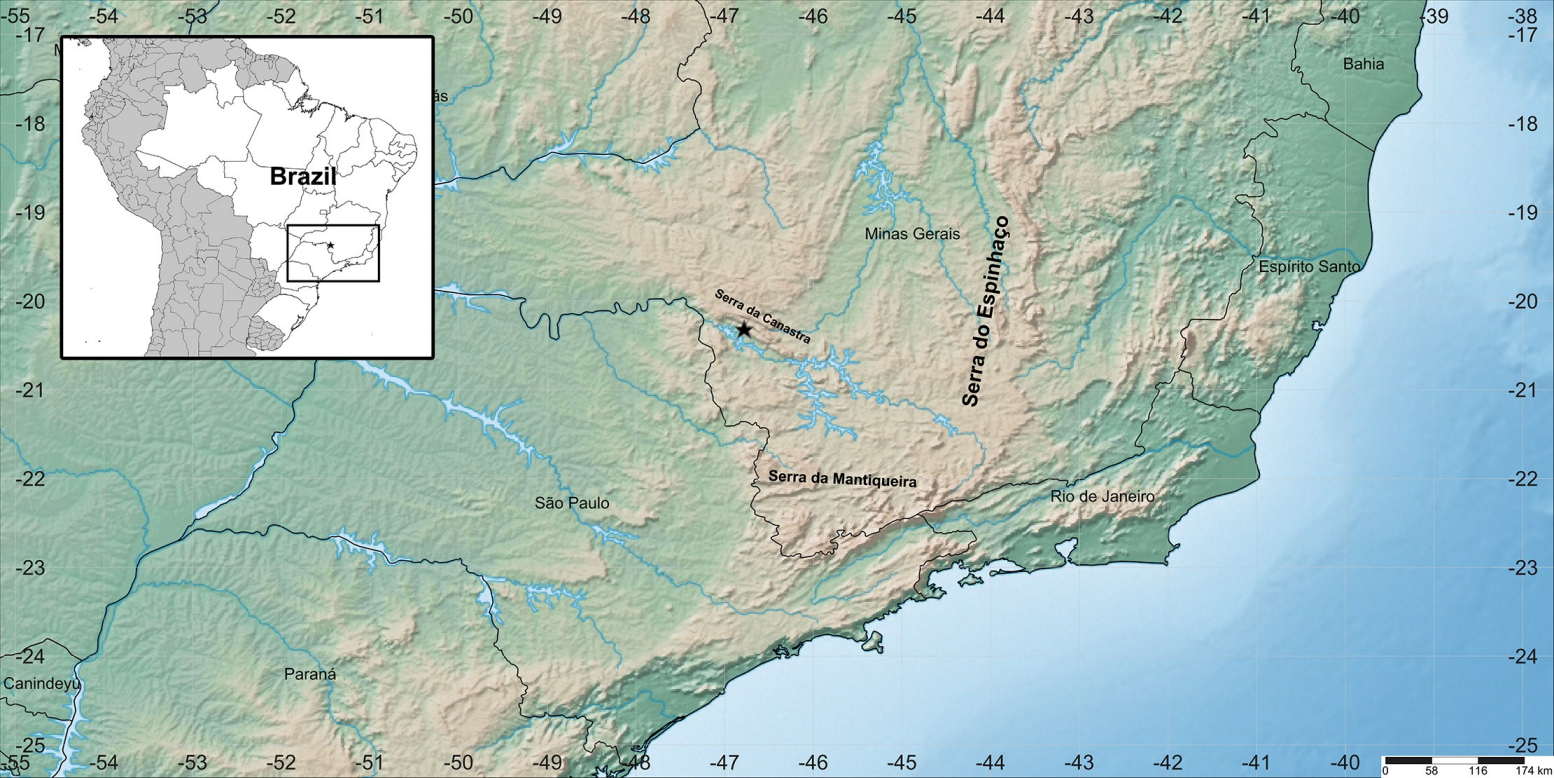
Distribution of *Genlisea hawkingii* in Serra da Canastra, Delfinópolis municipality in Minas Gerais, Brazil (Modified from https://www.simplemappr.net).

So far *Genlisea hawkingii* has only been found in Serra da Canastra, in the Delfinópolis municipality in Minas Gerais, Brazil (Fig 4).

### Etymology

The species epithet ‘*hawkingii’* was attributed as homage to the great English theoretical physicist and cosmologist, Stephen William Hawking, who died on March 14, 2018. We were impressed with his life’s trajectory and his outstanding discoveries in cosmology. He became a signpost not only for other scientists but for all people.

### Conservation status

The conservation status according to the IUCN [23] is Data Deficient (DD). The only known population of this species is currently found in Serra da Canastra near the Delfinópolis municipality (Minas Gerais, Brazil). The population occupied ~90 m^2^ and around 80 individuals were found. However, more populations may be found as this area has been poorly explored. This location suffers from an anthropogenic impact–cattle, and horses trampling, as it is located inside a farm near the boundaries of the Serra da Canastra National Park.

### Ecology and phenology

*Genlisea hawkingii* is a montane species (1,080–1,140 m) which grows among rocks, on shallow and sandy soils and also been near perennial water bodies such as streams and waterfalls. It is found within the altitudinal range of *G*. *flexuosa* (ca. 700–1,400 m), *G*. *exhibitionista* (1,000–1,400 m), *G*. *lobata* (1,000–1,722 m) and *G*. *violacea* [(680–) 900–1,950 m] [1,10]. Usually found associated with grasses and sedges (Poaceae and Cyperaceae, respectively), shrubs and other carnivorous plant species such as *Utricularia nana* A.St.-Hil. & Girard and *U*. *triloba* Benj., but not occurring sympatrically with other species of *Genlisea*. *G*. *hawkingii* is an annual species (personal observation during one complete year), as *G*. *exhibitionista*, *G*. *lobata* and *G*. *violacea*, in contrast to *G*. *uncinata* and *G*. *flexuosa* which are perennial plants [1,10]. The massive flowering and fruiting were observed in March and May.

### Species comparison

According to the subgeneric classification of *Genlisea* [1,10], *G*. *hawkingii* can be placed in the *Genlisea* subgen. *Tayloria* due to its bivalvate capsule dehiscence, which is curved downward in its fruit (Figs 1 and 2) and its Neotropical distribution.

*Genlisea hawkingii* resembles a robust *G*. *violacea* or *G*. *flexuosa*, except for the flower spur, which resembles *G*. *uncinata* (Table 2). However, in its vegetative state, *G*. *hawkingii* can be distinguished from its related species by having glabrous and dark green leaves, not green and with a few hairs. Regarding the reproductive organs, *G*. *hawkingii* is distinguished from *G*. *violacea* and *G*. *flexuosa* by the flowers, which have sepals with an acute apex and a long conical spur that is curved upward differently from *G*. *flexuosa* and *G*. *violacea* whose spurs are cylindrical, straight and are rarely curved downward, and are as long as the upper lip or slightly shorter. All the individuals found (~80 individuals) presented the same long conical and curved upward spur, therefore these characteristics are possibly stable for the new species. In addition, *G*. *hawkingii* only has glandular capitate hairs.

**Table 2:** Comparison of *Genlisea hawkingii*, *G*. *flexuosa*, *G*. *violacea* and *G*. *uncinata*. The measurements were taken from collected specimens and literature [1, 10]. “-” denotes missing data.

Characters	Species			
	*G*. *hawkingii*	*G*. *flexuosa*	*G*. *violacea*	*G*. *uncinata*
**Life span**	annual	perennial	annual	perennial
**Habit**	up to 30 cm	up to 60 cm	up to 25(35) cm	up to 80 cm
**Leaves**	green, dark green	pale green	pale green, dark green, or reddish	green, slightly succulent
	lamina obovate	lamina obovate to oblong	lamina obovate-spathulate to obovate	lamina obovate to transversely elliptical
	Glabrous	glabrous or adaxial and abaxial surface with sparse, long stalked hairs	glabrous	-
**Inflorescence**	lax raceme	lax raceme	lax raceme	dense raceme
	with 1–7 flowers	with up to 30 flowers	with 1-6(12) flowers	with up to 21 flowers
	scape, bracts, bracteoles, pedicels, calyx and ovary densely covered by exclusively glandular hairs	scape, bracts, bracteoles and calyx densely covered by glandular hairs and numerous simple eglandular hairs	scape, bracts, pedicels and ovary covered with glandular and few eglandular hairs, or by glandular hairs only	scape, bracts, bracteoles and pedicels densely covered by glandular and few eglandular hairs
**Pedicels**	curved downwards after anthesis for frutification	slightly prolonged and curved downwards after anthesis for frutification	reflexed and enlarged to 32 mm in fruit	strongly circinate in fruit
**Sepals**	lanceolate	lanceolate to obovate	oblong, ovate-lanceolate or elliptical	ovate, elliptical or oblong
	slightly curved, apex acute, pointed	apex obtuse to rounded	apex acute, pointed, rarely obtuse or emarginate	two lowermost sepals slightly curved
	densely covered exclusively with glandular hairs	-	covered with glandular and few eglandular hairs, or by glandular hairs only	densely covered exclusively with glandular hairs
**Corolla**	Bilabiate	bilabiate	bilabiate	bilabiate
	up to 10 mm long	up to 17 mm long	(5)7-12(16) mm long	up to 20 mm long
	upper lip cleft	upper lip broadly oblong, upper third deeply divided in to two divergent lobes	upper lip deeply bilobate, obcordate	upper lip entire, semicircular to very broadly ovate
	spur conical, perpendicular to the lower lip corolla, subulate towards the tip	spur cylindrical, straight or rarely slightly curved downwards near the apex, widening towards the tip	spur cylindrical, straight or sometimes slightly curved downwards near apex, widened towards apex	spur conical, from slightly constricted base
	apex curved	obtuse apex	apex rounded, obtuse or retuse	apex curved (uncinate), longer than lower corolla lip
	much longer than the upper lip of the corolla; usually longer than the lower corolla lip	much longer than the upper lip of the corolla	shorter than (rarely almost equaling) lower corolla lip	longer than pedicel and longer than lower corolla lip
	spur covered with glandular hairs	spur with short-stalked glandular hairs near the tip	spur densely covered with glandular hairs	spur densely covered with glandular hairs
	margins of corolla with glandular capitate hairs	corolla lower surface and margins (sub)glabrous	corolla margins (sub)glabrous or glandular	margins of corolla lobes densely covered with glandular hairs
**Capsule**	Globose	globose to broadly ovoid	globose	globose
	1.7-2(3) mm in diameter	3–3.5 mm in diameter	2-3(5) mm in diameter	ca. 3–4.5 mm in diameter
	covered with glandular hairs	covered with glandular hairs	covered with glandular hairs	covered with glandular hairs
**Seeds**	broadly angulate-ellipsoid to slightly prismatic	broadly angulate-ellipsoid to slightly prismatic	prismatic	ellipsoid
**Geographical distribution**	Brazil, Southern of Minas Gerais, in Serra da Canastra	Brazil, Northern of Minas Gerais State, in Serra do Espinhaço region	Brazil, broad distribution in Minas Gerais State and probably extinct in São Paulo State	Brazil, Bahia State, in Chapada da Diamantina

### Additional specimens examined (paratypes)

BRAZIL. Minas Gerais: Delfinópolis, Estrada da Casinha Branca, Fazenda Zé Antunes, Mata de Galeria, Solo hidromórfico, Planta com 0.3 m. Flores arroxeadas. 11 April 2002, *R*.*A*. *Pacheco*, *166* (HUFU!). Serra da Canastra, próximo da Casinha Branca, cerrado, solo arenoso entre rochas, próximo ao rio. Folhas verdes, eixo das inflorescências e cálices verdes, corolas arroxeadas com mácula amarela. 07 February 2019, *V*.*F*.*O*. *Miranda et al*. 2307 (JABU!); Serra da Canastra, próximo da Casinha Branca, cerrado, solo arenoso entre rochas na margem do rio. Folhas verdes, eixo das inflorescências e cálices verdes, corolas arroxeadas com mácula amarela. 16 April 2019, *V*.*F*.*O*. *Miranda et al*. 2359 (JABU!).

### Phylogenetic analyses

The combined gene analyses (*rps*16 + *mat*K) resulted in a matrix of 1,436 bp with 591 bp from the *rps*16 and 844 bp from the *mat*K fragments.

**Fig 5:**
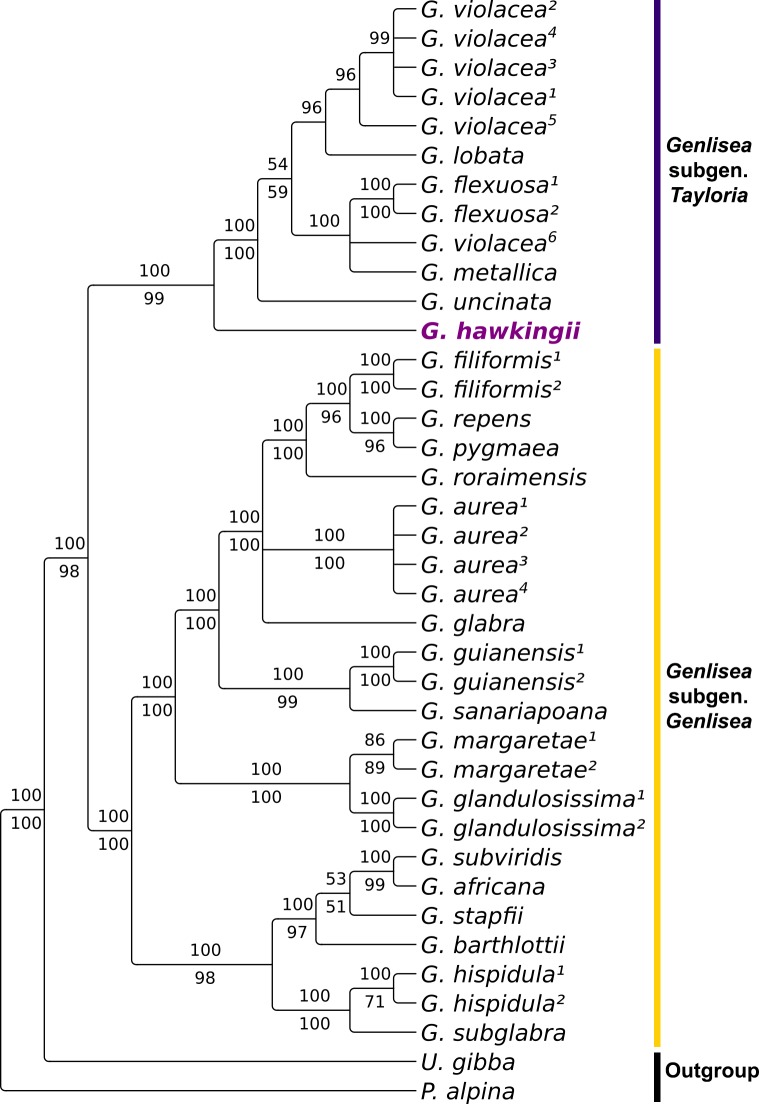
Phylogeny of the *Genlisea* species based on the Bayesian inference (BI) and maximum likelihood (ML) analyses of the combined dataset (*rps*16 + *mat*K). The numbers above and below the branches refer to the BI posterior probability and to the ML bootstrap support, respectively. The numbers beside the species names are references to the different specimens/populations and follow the numbers in Table 1.

The phylogenetic position of the new *Genlisea hawkingii* species, as it is presented in this study was strongly supported to be in *G*. subgen. *Tayloria* according to the BI and ML analyses (Fig 5). Moreover, despite having morphological characteristics that are similar to *G*. *flexuosa* and *G*. *violacea*, it is an early branching species to the other species of *G*. subgen. *Tayloria*, and is followed by *G*. *uncinata*, which is similar to *G*. *hawkingii* in a few morphological characteristics such as the curved spur (Table 2). Variations in flower morphology, including the spur shapes and sizes, can affect the pollination success [24], therefore the curved spur found in *Genlisea* can be explained as a possible adaptation to different pollinators [7,25]. However, further studies regarding the pollinator fauna are needed to clarify the pollination biology for the species of *Genlisea*.

Considering that *G*. *hawkingii* and *G*. *uncinata* present curved spur and for the other species of *G*. subgen. *Tayloria* this structure is straight (in *G*. *metallica* it can be slightly curved downwards near the apex) [7], two hypotheses can be explored by optimizing the transformations of this character in the tree, and having both accepted with the parsimony approach (ACCTRAN and DELTRAN [26]) since both hypotheses assume the same number of steps (two in this case). Thus, the curved spur can be the effect of parallelism for *G*. *hawkingii* and *G*. *uncinata*, as a result of two autapomorphies, or this state can be assumed to be a synapomorphy to the *G*. subgen. *Tayloria* clade with the posterior reversion to the *G*. *violacea-G*. *lobata-G*. *flexuosa-G*. *metallica* clade.

*G*. *hawkingii* is shown to be an early-branching lineage considering the *G*. subgen. *Tayloria* clade (Fig 5), thus contradicting the assumption that *G*. *uncinata* could be a relict lineage exhibiting plesiomorphic states for some characters [7]. For instance, the entire upper lip of *G*. *uncinata* can be the result of a reversion of this character, considering that *G*. *hawkingii* and other members of *G*. subgen. *Tayloria* (except *G*. *uncinata*) show a bilobate upper lip [1,10].

Even with the presented hypothesis based on two markers (Fig 5), a more complete analysis with additional species, populations and DNA sequences could bring a robust and comprehensive hypothesis to the phylogeny of *G*. subgen. *Tayloria*. For example, the morphological diversity between populations of *G*. *violacea* and its paraphyly in phylogenetic hypotheses (Fig 5) [7] suggest that it represents a complex of different species and, therefore, further studies based on morphological and molecular data are necessary to address this issue.

Therefore, a phylogenomic approach based on plastidial [27] and mitochondrial genomes is under construction (Silva et al., in preparation) and possibly will result in a more robust phylogeny for the group.
